# The effect of oxytocin vaginal gel on vaginal atrophy in postmenopausal women: a randomized controlled trial

**DOI:** 10.1186/s12905-020-00935-5

**Published:** 2020-05-19

**Authors:** Ilnaz Zohrabi, Parvin Abedi, Somayeh Ansari, Elham Maraghi, Nader Shakiba Maram, Gholamreza Houshmand

**Affiliations:** 1 Menopause Andropause Research Center, Midwifery Department, Nursing & Midwifery School, Ahvaz Jundishapur University of Medical Sciences, Golestan Ave, Ahvaz, Iran; 2 Department of Biostatistics and Epidemiology, Faculty of Public Health, Ahvaz Jundishapur University of Medical Sciences, Ahvaz, Iran; 3 Pharmaceutics Department, Nanotechnology Research Center, Ahvaz Jundishapur University of Medical Sciences, Ahvaz, Iran; 4 Department of Pharmacology, School of Medicine, Mazandaran University of Medical Sciences, Sari, Iran

**Keywords:** Oxytocin, Vaginal atrophy, Postmenopausal women

## Abstract

**Background:**

Around 90% of postmenopausal women are suffering from vaginal atrophy. This study aimed to evaluate the effect of oxytocin vaginal gel on vaginal atrophy among postmenopausal women.

**Methods:**

This was a randomized controlled trial that was conducted on 96 postmenopausal women who suffered from vaginal atrophy. The inclusion criteria were: literate women, age 40–60, at least 1 year passed from their last menstrual period or the level of FSH > 40 IU, monogamous women with the sexual relationship. Women in the intervention group, requested to use one applicator of 400 IU oxytocin gel per night and women in the placebo group used placebo each night. The subjective symptoms of vaginal atrophy, vaginal PH, maturation index were measured before and after the intervention.

**Results:**

The number of superficial cells was increased significantly in the oxytocin group compared to placebo (38.7 ± 7.18 vs. 3.69 ± 2.76, *p* = 0.0001), while the number of parabasal cells was decreased significantly in the oxytocin compared to placebo after the intervention. The improvement of the maturation index was more dominant in the oxytocin group (increased from 7.76 ± 4.68 to 52.48 ± 7.54) in comparison to the placebo group (increased from 8.58 ± 4.35 to 13.25 ± 5.06). The PH of the vagina decreased significantly in the oxytocin group in comparison to the placebo group (*p* = 0.0001). After 8 weeks, 88.6 and 7.1% of women in the oxytocin and placebo groups did not show the severe symptoms of vaginal atrophy (*p* = 0.001).

**Conclusion:**

The results of this study showed that eight- week intervention with oxytocin vaginal gel (400 IU) could significantly improve the vaginal maturation index, subjective symptoms of vaginal atrophy and reduce the PH of the vagina. Using this medication in women who have a contraindication for hormone therapy is recommended.

**Trial registration:**

IRCT20160602028220N2.

## Background

Vulvovaginal atrophy (VVA) is a phenomenon that happens mostly after menopause and affects around 90% of postmenopausal women [1]. Only a quarter of women with VVA seek treatment [2]. Women with VVA may experience symptoms such as dryness, irritation, itching, burning, and dyspareunia [3]. The VVA is often due to the estrogen deficiency after menopause and may negatively affect the quality of life [1]. The low level of estrogen decreases the blood flow to the vulvovagina and making the tissue of the vagina to be thinner and prone to bleeding and infection. On the other hand, the low level of estrogen may decrease the number of lactobacilli and causing increased PH of the vagina [4].

The first- line treatment of VVA is a continuous sexual relationship, using non-hormonal over the counter vaginal lubricant and lifestyle change [5]. Women can use vaginal lubricants before each sexual relationship or vaginal lubricant with long-term impact in which they should use regularly (two or three times a week) [6].

The systemic and local estrogen is recommended for the treatment of VVA in postmenopausal women, in which low dose local vaginal cream preferred [7]. According to the US Department of Health and Human Services, Food and Drug Administration, estrogen is recommended for the treatment of vasomotor symptoms and vulvovaginal atrophy in postmenopausal women [8]. Although hormone therapy may alleviate the short-term and long term complications of menopause such as hot flashes, night sweats, mood swings, and osteoporosis, it may increase the risk of breast and endometrial cancer [9].

Because of the adverse effects of HRT, some women prefer not to use this method for alleviating vaginal atrophy, and they tend to use non-hormonal medication for this matter [10]. Studies showed that some herbs and vitamins such as fennel [11], vitamin E [12], vitamin D [13], could reduce vaginal atrophy in postmenopausal women. Oxytocin is a hormone and a neurotransmitter of the brain and its main role is the flow of milk from the breasts [14]. Oxytocin also promotes positive social behavior, stress regulation, [15] and female sexual arousal [16]. The role of oxytocin on vaginal atrophy was examined first by Al-Saqi et al., and they found that oxytocin is a useful means for reducing vaginal atrophy in postmenopausal women [17]. Recently another study examined the effect of oxytocin vaginal gel on vaginal atrophy and the results showed that oxytocin could significantly reduce the vaginal atrophy after 30 days of the intervention [18]. There are only a few studies that evaluated the impact of vaginal gel of oxytocin on vaginal atrophy that one of them followed participants for 4 weeks and the other for 7 weeks. As using non-hormonal methods for vaginal atrophy needs more investigation, therefore, we intended to evaluate the effect of intra-vaginal oxytocin gel on vaginal atrophy in postmenopausal women in a longer period.

## Methods

This was a randomized controlled trial that was conducted on 96 postmenopausal women who suffered from vaginal atrophy in two health centers in Ahvaz, Iran. The inception date of this study was in April 2018 and the completion date was September 2018. The protocol of this study was approved by the Ethics Committee of Ahvaz Jundishapur University of Medical Sciences (Ref No: IR.AJUMS.REC.1396.720). This study was registered in the Iranian Registry for Clinical Trials (Ref No: IRCT20160602028220N2). This study adheres to CONSORT guidelines for a randomized controlled trial (CONSORT checklist, supplementary material). The inclusion criteria were as follow: literate women, age 40–60, at least 1 year passed from their last menstrual period or the level of FSH > 40 IU, monogamous women with a sexual relationship. Women with following criteria were excluded from the study: vaginal infection, women who used hormone replacement therapy, any undiagnosed genitalia diseases, smoker’s women, the body mass index more than 30 kg/m^2^, vaginal bleeding or spotting, any breast diseases with unknown cause, using vaginal lubricant at least 15 days before the intervention. All women provided written informed consent before data collection. Menopause was confirmed by results of FSH in some participants, but in most participants, the menopause were confirmed if the menses was ceased for 1 year.

### Sample size calculation

We used the following formula with considering Al-Saqi et al’s study, [17] β = 0.9, α = 0.05, S1^2^ = S2^2^ = 1.795, d = 1.0035, and considering 25% attrition the total sample size for each group was calculated to be 48.

$$ \mathrm{n}=\frac{{\left(Z1-\frac{\alpha }{2}+Z1-\beta \right)}^2\left({s}_1^2+{s}_2^2\right)}{{\left(\mathrm{d}\right)}^2} $$

### Drug preparation

Oxytocin powder was purchased from Caspian Company at 0.08% and stored at 2 to 8 °C, away from light. For the vaginal gel preparation, at first, the 2% sodium carboxymethyl cellulose was added to the 20% propinyl glycol gradually when it was stirring. Then the boiled water containing 0.2% methylparaben was added and stirred until it was cool. Then 2 g oxytocin powder in the distilled water residue (39%) was added and stirred to form the oxytocin gel. The same steps were taken to make a placebo, except the oxytocin powder was not added. Oxytocin gel and placebo were placed in similar tubes and the pharmacist assigned codes to these tubes. The researcher and the participants were not aware of the content of each tube.

### Recruitment

Eligible women were placed in the lithotomy position and the vagina was assessed regarding infection and any abnormal discharge. Women who did not have any of these symptoms were considered for intervention. Then women were evaluated regarding vaginal signs and symptoms of vaginal atrophy such as dryness, pallor, dyspareunia, redness, inflammation, and vulvovaginal erosion. The subjective symptoms of vaginal atrophy were assessed using the self- reported scale of burning, itching, feeling of dryness and dyspareunia. The severity of each symptom was determined by the patient and then an appropriate score was applied as follows: the absence of any symptoms received zero, mild symptoms received 1, moderate and intense symptoms received 2 and 3 respectively. The sum of scores was calculated for each woman. If the total score was < 5, we considered the patient as vaginal atrophy.

For measuring vaginal PH, a disposable speculum (Bekr brand) was inserted into the vagina and a sample from the posterior fornix was taken using a cotton swab (Neeva) and placed in the lamella and fixed with fixator (Patofix) and sent to a reference laboratory for vaginal maturation index evaluation (VMI). The vaginal PH was measured using a PH gauge paper (Merck, Germany). The paper tape was contacted to the vaginal wall and kept for 1 minute and the corresponding strip was compared to the PH bar and the number recorded in the checklist. If the vaginal atrophy (signs and subjective symptoms) was confirmed, women were recruited for the intervention.

### Randomization

Eligible women were randomly assigned in two groups of vaginal gel of oxytocin or placebo using block randomization with a block size of 4 and the ratio of 1:1. For allocation concealment, each woman received a code and all codes were kept in an opaque envelope until the time of intervention. The researcher and the participant were not aware of the groups and the intervention that they received. The oxytocin and placebo were placed in similar tubes and coded by the pharmacist. None of the researchers or participants was aware of real treatment.

### Measurement

The following forms were used for data collection. A demographic questionnaire was used for recording characteristics such as age, education level, and economic situation. A checklist was used for recording signs and symptoms of vaginal atrophy and PH. Also, a checklist was used for recording the subjective symptoms of vaginal atrophy. Except for the demographic questionnaire that completed at baseline, other checklists were completed at baseline, two and eight weeks after intervention. The validity and reliability of all questionnaire and checklist were assessed using content validity. The body mass index was calculated by weight (kg)/height (m^2^) and self-reported by participants. The maturation index and vaginal PH were measured at baseline and 8 weeks after the intervention, while the subjective symptoms of vaginal atrophy were measured at baseline, two and eight weeks after intervention.

### Intervention

Women in the intervention group requested to use oxytocin gel (400 IU) and women in the placebo group used placebo vaginal gel for 8 weeks. An applicator contains 4 g and participants requested to filled-up one- quarter of each applicator per night and continue to use for eight consecutive weeks.

### Statistics

All data entered SPSS version 22. The normal distribution of data was assessed using the Shapiro–Wilk test. The continuous data were analyzed using independent t-test, while the categorical data was analyzed using the chi-square test. The Mann-Whitney test was used for comparing dyspareunia between two groups. For variables with more than two measurements, the Generalized Estimating Equations (GEE) was used. *P* < 0.05 was considered significant.

## Results

**Fig. 1:**
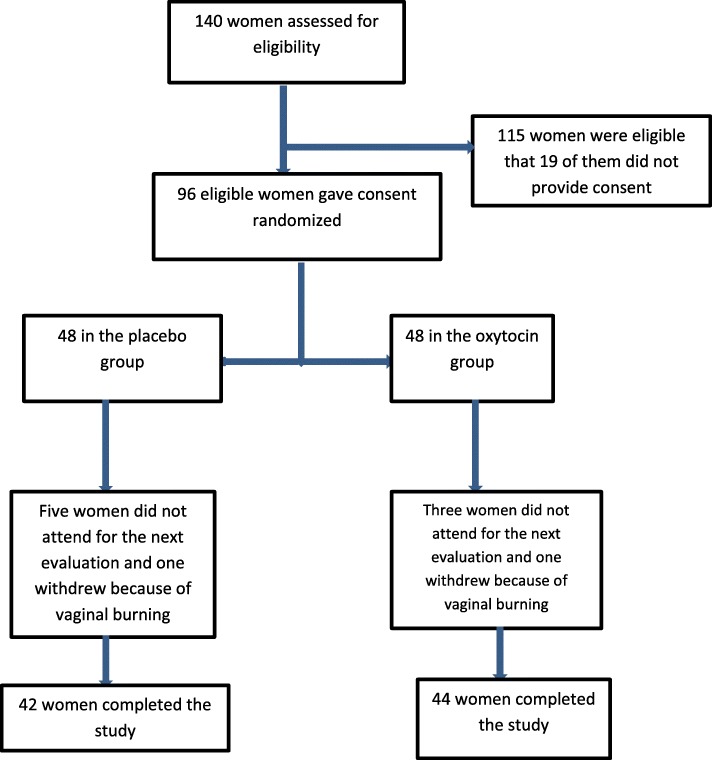
Flow diagram of recruitment and retention of participants in the study

A total number of 98 women were recruited in this study; however, four women in the placebo and five women in the oxytocin group withdrew the study. The reasons for drop-out are listed in Fig. 1. Table 1 shows the socio-demographic characteristics of participants in two groups of oxytocin and placebo. The mean age of women was 54.18 and 54.1 in the oxytocin and placebo groups. Most women in two groups experienced menopause around the age of 50 and most of the women were overweight. The frequency of coitus was low and in both groups was around three per month. Most of the women had primary education and were categorized in the moderate level regarding economic situation.

**Table 1:** Socio-demographic characteristics of participants in the oxytocin and placebo groups

Variables	Oxytocin *n* = 44	Placebo *n* = 42	*P* value
	Mean ± SD		
Age(y)	54.18 ± 3.31	54.1 ± 3.68	0.98
Age of menopause (y)	50 ± 2.16	50.38 ± 2.59	0.51
Years passed from menopause (y)	4.13 ± 2.01	3.78 ± 2.33	0.249
Body mass index(kg/m^2^)	28.5 ± 1.54	28.8 ± 1.49	0.26
Coitus per month	2.75 ± 1.34	2.38 ± 0.88	0.27
	N(%)		
**Education**			
Primary	20 (45.5)	14 (33.3)	0.46
Secondary	13 (29.5)	17 (40.5)	
Diploma and higher	11 (25)	11 (26.2)	
**Economic situation**			
Weak	17 (38.5)	16 (38.1)	0.92
Moderate	21 (47.7)	19 (45.2)	
Good	6 (13.6)	7 (16.7)	
**Job**			
Housewife	2 (4.5)	1 (2.4)	0.58
Employee	42 (95.5)	41 (97.6)	

Table 2 shows the mean of superficial, intermediate and parabasal, maturation index and PH of vagina before and after the intervention. The number of superficial cells was increased significantly in the oxytocin group compared to placebo (38.7 ± 7.18 vs. 3.69 ± 2.76, *p* = 0.0001). The number of the intermediate cell increased, while the number of parabasal cells was decreased significantly in the oxytocin compared to placebo after the intervention. The improvement of the maturation index was more dominant in the oxytocin group (increased from 7.76 ± 4.68 to 52.48 ± 7.54) in compare to placebo group (increased from 8.58 ± 4.35 to 13.25 ± 5.06). The PH of the vagina decreased significantly in the oxytocin group in comparison to the placebo group after 8 weeks of treatment (*p* = 0.0001).

**Table 2:** The maturation index and vaginal PH before and after intervention in two groups of oxytocin and placebo

Variables		Oxytocin N = 44	Placebo N = 42	*P* value between groups
	Mean ± SD			
**Superficial cells**	Before	0.59 ± 1.38	0.35 ± 0.79	0.34
	After	38.7 ± 7.18	3.69 ± 2.76	0.0001
*P value within group*		0.0001	0.0001	
**Intermediate cells**	Before	14.54 ± 8.53	16.6 ± 8.59	0.26
	After	27.56 ± 5.77	19.07 ± 8.56	0.0001
*P value within group*		0.0001	0.0001	
**Para basal cells**	Before	84.8 ± 9.18	83.02 ± 8.57	0.35
	After	33.95 ± 9.17	77.23 ± 8.97	0.0001
*P value within group*		0.0001	0.0001	
**Vaginal maturation index**	Before	7.76 ± 4.68	8.58 ± 4.35	0.4
	After	52.48 ± 7.54	13.25 ± 5.06	0.0001
*P value within group*		0.0001	0.0001	
**Vaginal PH**	Before	6.01 ± 0.75	6.19 ± 0.79	0.28
	After	4.51 ± 0.51	6.07 ± 0.73	0.0001
*P value within group*		0.0001	0.13	

Table 3 shows the subjective symptoms of vaginal atrophy before and after the intervention. As evident from this table, at baseline, most women in two groups had severe symptoms. Two weeks after the intervention, 20.5 and 4.8% in the oxytocin and placebo groups were free of severe subjective symptoms of vaginal atrophy (*p* = 0.001). After 8 weeks of intervention, 88.6 and 7.1% of women in the oxytocin and placebo groups did not show the severe symptoms of vaginal atrophy (*p* = 0.001).

**Table 3:** The subjective symptoms of vaginal atrophy and dyspareunia before and after intervention in the oxytocin and placebo groups

Variables	Dyspareunia	Oxytocin n = 44	Placebo n = 42	*P* value using Mann Whitney U test	*P* value using GEE
		N(%)			
Before intervention	Negative	2 (4.5)	2 (4.8)		
	Mild	13 (9.5)	12 (28.6)	0.514	
	Moderate	14 (31.8)	9 (21.4)		
	Severe	15 (34.1)	19 (45.2)		
Two weeks after intervention	Negative	9 (20.5)	2 (4.8)		
	Mild	19 (43.2)	13 (31)	< 0.001	< 0.0001
	Moderate	13 (29.5)	9 (21.4)		
	Severe	3 (6.8)	18 (42.9)		
Eight weeks after intervention	Negative	39 (88.6)	3 (7.1)		
	Mild	5 (11.4)	19 (45.2)	< 0.001	
	Moderate	0	13 (31)		
	Severe	0	7 (16.7)		

The Mann Whitney U test was used to detect differences between two groups

Table 4 demonstrates the mean of subjective symptoms score in two groups of oxytocin and placebo. The scores of subjective symptoms were decreased significantly in the oxytocin group (from 6.2 ± 2.67 to 0.38 ± 0.96) compared to the placebo group (from 6.54 ± 2.82 to 4.9 ± 2.9) (*p* = 0.0001). One woman in each group of oxytocin or placebo withdrew the study because of vaginal burning.

**Table 4:** Comparison of scores of subjective symptoms of vaginal atrophy in two groups of oxytocin and placebo

Variables	Oxytocin n = 44	Placebo n = 42	*P* value
	Mean ± SD		
Before intervention	6.2 ± 2.67	6.54 ± 2.82	0.56
After intervention	0.38 ± 0.96	4.9 ± 2.9	0.0001
*P* value	0.0001	0.0001	

## Discussion

This study was designed to evaluate the effect of oxytocin vaginal gel on vaginal atrophy in postmenopausal women. Our results revealed that oxytocin vaginal gel could significantly increase the superficial cells after 2 months of intervention, and also the vaginal maturation index improved significantly in the oxytocin compared to the placebo groups. Oxytocin is a hormone that has many receptors in the endometrium and these receptors can be stimulated for the onset of labor [19]. Although we could not find a study that shows the effect of oxytocin on cell maturation, we rely on the results of some studies that evaluated the effect of oxytocin on endometrial cells. Roshangar et al. found that oxytocin has a stimulating effect on endometrium cells in mice [20]. Also, Al-Eknah et al., in their study found that oxytocin injected to goats could significantly reduce the level of progesterone in the plasma and also could change the content of protein, acid and alkaline phosphatase in the cervical mucus [21]. There is some evidence that oxytocin may improve the growth of epithelium and endothelial cells [22]. Oxytocin also may improve the blood circulation to the vagina and increase the lubrication of the vagina [23]. Roshangar et al. in their study found that injection of 1 IU/gr oxytocin could significantly increase the endometrial thickness in the rat after 48 h [24].

In the present study, all of the subjective symptoms of vaginal atrophy significantly improved in the oxytocin group compared to the placebo. The effect of oxytocin on vaginal atrophy in postmenopausal women also was investigated by Jonasson et al. (2011) and the results showed that oxytocin could significantly reduce the symptoms of vaginal atrophy such as vaginal dryness, pain, itching, discomfort and dyspareunia after one- week intervention. The level of circulating estrogen has remained unchanged in comparison to the baseline. The authors concluded that this study should be repeated with a larger sample size [25]. These results are in line with our study.

Our results showed that the PH of the vagina was decreased significantly after 8 weeks of intervention in the oxytocin group compared to the placebo. A study assessed the effect of prostaglandin F2α and oxytocin on vaginal microflora in *awassi* sheep ewes and concluded that while prostaglandin F2α decreased the amount of vaginal microflora, the microflora remained unchanged in the oxytocin [26].

Al-Saqi et al. (2015) conducted a study on 64 postmenopausal women and assessed the effect of oxytocin vaginal gel (400 IU, 100 IU or placebo) on vaginal atrophy. Their results showed that after 7 weeks of intervention the cytology of the vagina improved and the number of superficial cells was increased and the PH of the vagina decreased. The thickness of the endometrium did not change [17]. Torky et al., conducted a study on 140 women (70 in the oxytocin and 70 in the placebo groups) and used 600 IU oxytocin gel or placebo to relief the vaginal atrophy and followed patients for 1 month. Their results showed that all subjective and objective symptoms of vaginal atrophy decreased significantly in the oxytocin group [18]. These results are similar to what we found in our study.

### Strengths and limitations of the study

This was a double- blind randomized controlled trial with the eight- week intense follow-up. All measurements and follow-ups were conducted by one researcher. One of the limitations of this study is; we did not assess the level of blood estrogen of participants. However, other studies showed that oxytocin did not affect the blood estrogen level [18]. In this study, because of financial constraints, we were not able to prepare disposable applicators and used one applicator to transfer oxytocin gel or placebo into the vagina. Participants were taught to fill a quarter of applicator to reach the level of 1 g. This may cause errors and affected the results. In this study we followed participants until 8 weeks. Because vaginal atrophy is a chronic process and requires long-term treatment, other studies with a longer follow-up period may yield better results.

## Conclusion

The results of this study showed that 8 weeks of intervention with oxytocin vaginal gel (400 IU) could significantly improve the vaginal maturation index, subjective symptoms of vaginal atrophy and reduce the PH of the vagina. Using this medication in women who have a contraindication for hormone therapy is recommended.

## Data Availability

Data of this study will be available upon the request from the corresponding author.

## Abbreviations

VVA: Vulvovaginal atrophy
VMI: Vaginal maturation index
FSH: Follicle stimulating hormone
IU: International unit
HRT: Hormone replacement therapy
PH: Potential hydrogen
CONSORT: Consolidated standards of reporting trials
GEE: Generalized estimating equations
